# The Impact of an SGLT2 Inhibitor versus Ursodeoxycholic Acid on Liver Steatosis in Diabetic Patients

**DOI:** 10.3390/ph15121516

**Published:** 2022-12-05

**Authors:** Sahar H. Elhini, Engy A. Wahsh, Ahmed A. Elberry, Nadia F. El Ameen, Ahmed Abdelfadil Saedii, Shereen Mahmoud Refaie, Asmaa A. Elsayed, Hoda M. Rabea

**Affiliations:** 1 Diabetes and Endocrinology Unit, Internal Medicine Department, Faculty of Medicine, Minia University, Minia 61111, Egypt; 2 Clinical Pharmacy Department, Faculty of Pharmacy, October 6 University, Giza 12525, Egypt; 3 Clinical Pharmacology Department, Faculty of Medicine, Beni-Suef University, Beni-Suef 62551, Egypt; 4 Department of Pharmacy Practice, Pharmacy Program, Batterjee Medical College, Jeddah 21442, Saudi Arabia; 5 Radiology Department, Faculty of Medicine, Minia University, Minia 61111, Egypt; 6 Clinical Pathology Department, Faculty of Medicine, Minia University, Minia 61111, Egypt; 7 Department of Biomedical Sciences, College of Medicine, King Faisal University, Hofuf 31982, Saudi Arabia; 8 Clinical Pharmacy Department, Faculty of Pharmacy, Sohag University, Sohag 82511, Egypt; 9 Clinical Pharmacy Department, Faculty of Pharmacy, Beni-Suef University, Beni-Suef 62551, Egypt

**Keywords:** ursodeoxycholic acid, empagliflozin, type 2 diabetes, NAFLD, MRI-PDFF

## Abstract

Non-alcoholic fatty liver disease (NAFLD) is related to metabolic syndrome via insulin resistance, where preventing disease progression is crucial in the management process. The study included 240 NAFLD patients with type 2 diabetes who were randomly allocated into empagliflozin 25 mg (EMPA group), ursodeoxycholic acid 250 mg (UDCA group), or the control group (placebo). The study outcomes included: changes in liver fat content (LFC; %) (utilizing the Dixon-based MRI-PDFF approach), liver enzymes, lipid and glycemic profiles, FIB-4 index, and non-alcoholic fatty liver score (NFS). All endpoints were assessed at baseline and after 6 months. EMPA outperformed UDCA and placebo in decreasing LFC (−8.73% vs. −5.71% vs. −1.99%; *p* < 0.0001). In post-treatment ultrasound images and MRI-PDFF calculations, more patients had normal fatty liver grade (no steatosis or LFC < 6.5%) with EMPA compared to UDCA. EMPA and UDCA showed significant regression in the FIB-4 index (−0.34 vs. −0.55; *p* = 0.011) and NFS scores (−1.00 vs. −1.11; *p* = 0.392), respectively. UDCA achieved higher reductions in insulin resistance than EMPA (*p* = 0.03); however, only EMPA significantly increased beta-cell function (54.20; *p* = 0.03). When exploring the differences between the two drugs, EMPA was better in decreasing LFC (%), while UDCA achieved higher reductions in liver fibrosis scores. Both showed a similar safety profile in managing liver steatosis.

## 1. Introduction

Non-alcoholic fatty liver disease (NAFLD) recognized as the presence of 5% or more fat accumulation in hepatocytes without hepatocellular injury determined by biopsy. The definite diagnosis for this disease is a matter of exclusion from other causes of secondary liver steatosis [1,2].

NAFLD is a complicated and multifactorial disorder affecting several organs and pathways. It can progress to liver fibrosis and cirrhosis. Globally, non-alcoholic fatty liver (NAFL) is a significant health burden linked to metabolic syndrome (Mets) and an increased risk of several renal, cardiovascular, and endocrine diseases. The emergency of such a multifaceted systemic disease demonstrates the need for a safe and effective treatment [3].

Most guidelines restricted pharmacological therapy to progressive non-alcoholic steatohepatitis (NASH), early-stage NASH with risk factors for disease progression (age > 50 years and Mets), and active NASH with necro-inflammation. Currently, the Food and Drug Administration (FDA) has not approved any drug for NAFLD treatment. Yet, all guidelines carefully outweigh any medication prescribed specifically for NAFLD in terms of benefits and safety [4].

Sodium-dependent glucose transporter 2 (SGLT2) inhibitors improve several aspects of Mets. Empagliflozin (EMPA) is an oral hypoglycemic drug that inhibits SGLT2 [5,6]. Preclinical studies on animal models showed that EMPA reduced liver steatosis through attenuating inflammation, oxidative damage, and dysregulated hormone secretion [7,8]. The EMPA-REG outcome study reported that patients on EMPA had lower blood sugar levels and improved hepatic lipid content, liver enzymes, and liver stiffness [9]. Additionally, previous studies on patients with/without diabetes highlighted the beneficial role of EMPA on liver steatosis [7,10,11,12].

Ursodeoxycholic acid (UDCA) is a bile acid derivative with anti-inflammatory, antioxidative, and anti-apoptotic properties [13]. Clinical studies of UDCA monotherapy on NAFLD features had conflicting findings [14], with higher doses (23–35 mg/kg/d) suggesting slightly beneficial outcomes [14,15]. UDCA failed to improve fibrosis scores in a recently published trial [16]. In all guidelines, UDCA is not recommended for NASH, but it could be a potential therapeutic target for NAFLD [15,17]. Therefore, we aimed to assess the differences between empagliflozin and ursodeoxycholic acid in terms of safety and efficacy as add-on therapy in regressing LFC and fibrosis in type 2 diabetic patients with NAFLD.

## 2. Results

### 2.1. Description of Study Cohort

**Figure 1:**
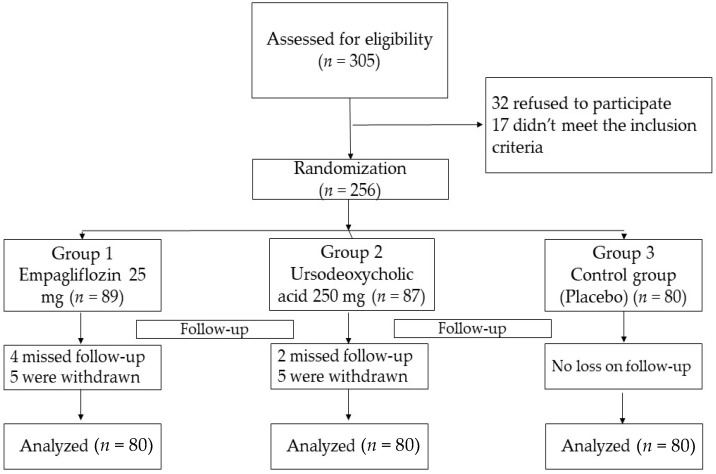
Flow chart of the study cohort.

From December 2020 to December 2021, 256 T2DM patients with NAFLD were randomly assigned to either study group. However, only 240 patients completed the trial (80 patients in each group). Ten patients withdrew after enrollment (due to scheduling problems or long distances), and six missed the follow-up visits (Figure 1).

All study subjects had a diabetes history ranging from one to ten years, mostly less than five years. The study groups exhibited similar demographic data (Supplementary Table S1). Comparisons between baseline clinical data of study groups are presented in Supplementary Table S2.

Both drugs were well tolerated, and fifteen patients (18.75%) reported adverse events with empagliflozin therapy. Ten females (12.5%) reported urinary tract infections and were referred to the Urology clinic, and five patients (6.25%) experienced recurrent urinary tract infections leading to drug discontinuation.

### 2.2. Effects on Demographic Characteristics and Other Biochemical Parameters

Table 1 summarizes the changes in different biochemical parameters in study groups.

**Figure 2:**
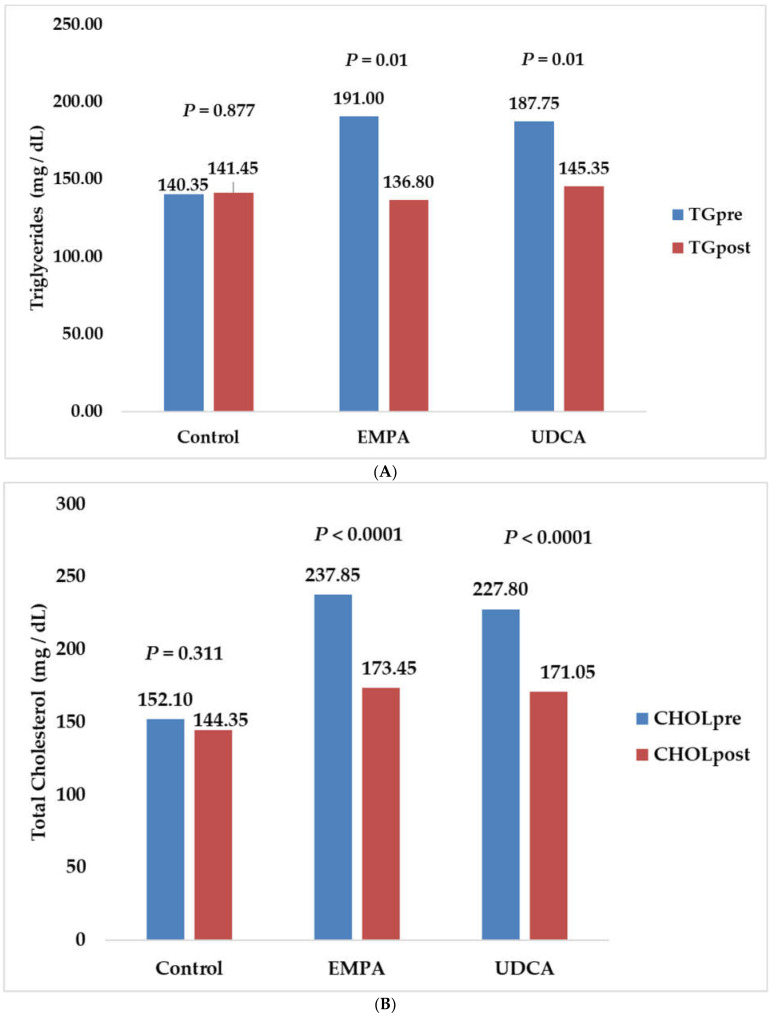

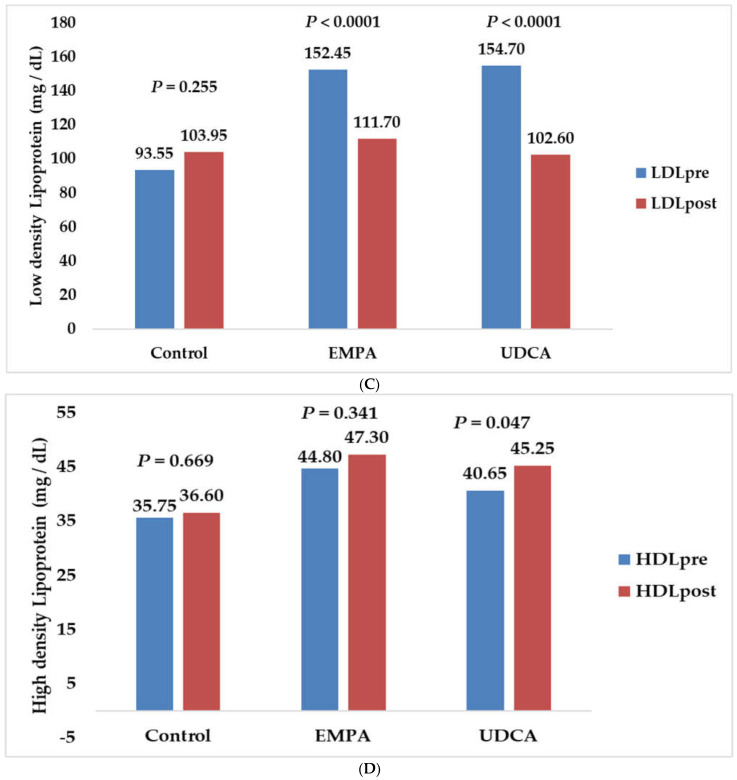
Changes in lipid profiles in study groups after six months. (**A**) Changes in serum triglycerides in study groups; (**B**) Changes in total cholesterol in study groups; (**C**) Changes in serum low-density lipoprotein in study groups; (**D**) Changes in high-density lipoprotein in study groups.

The EMPA and UDCA groups significantly reduced SBP (−5.50 mmHg; *p* = 0.01, −4.50 mmHg; *p* = 0.01, respectively), and DBP (−5.75; *p* < 0.0001 and −3.50; *p* = 0.01, respectively). The placebo group experienced significantly lower levels of SBP (−8.00; *p* = 0.001). Additionally, EMPA and UDCA significantly lowered BMI and waist-to-hip ratio. Empagliflozin had a higher reduction than UDCA in serum triglycerides and total cholesterol. However, only UDCA positively impacted HDL (3.01; *p* = 0.047) (Figure 2).

### 2.3. Effects on Liver Steatosis

Both drugs significantly decreased liver enzymes and sustained these changes over the study period. (Table 1 and Supplementary Figure S1) Twenty-five patients (31.25%) with EMPA therapy and eight (10%) with UDCA revealed grade 0 (no steatosis) on ultrasound post-treatment images. However, MRI-PDFF post-treatment images ruled out liver steatosis (LFC <6.5%) in fifteen (18.75%) patients with EMPA and eight (10%) with UDCA. 

EMPA, UDCA, and placebo groups significantly reduced LFC (%) (−8.73 vs. −5.71 vs. −1.99; *p* < 0.0001). Additionally, the LFC (%) changes in the EMPA vs. UDCA group were statistically significant (−3.02; *p* = 0.002). In full liver fat fraction mapping, EMPA significantly reduced LFC (%) in all liver segments, while UDCA achieved significant reductions in segments IVb, V, and VIII (Table 2).

**Figure 3:**
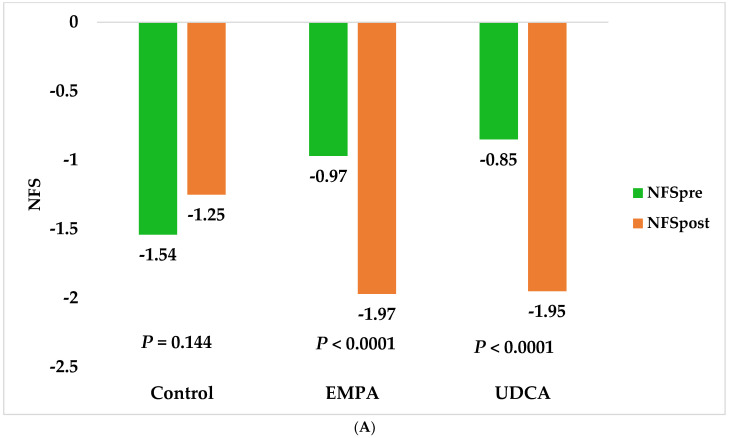

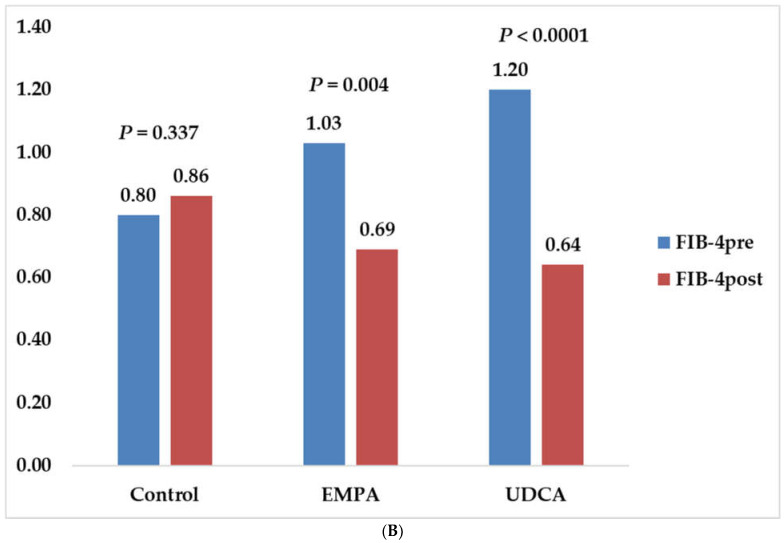
Changes in fibrosis indices in study groups after six months. (**A**) Changes in non-alcoholic fatty liver score. (**B**) Changes in the FIB-4 index.

Moreover, UDCA and EMPA significantly decreased NFS (−1.11; *p* < 0.0001 vs. −1.00; *p* < 0.0001, respectively) and FIB-4 index (−0.55; *p* < 0.0001 vs. −0.34; *p* = 0.004, respectively) (Figure 3).

### 2.4. Correlation Analysis

A correlation study was performed using Pearson correlation between the changes in LFC (%) values and the changes in the following parameters: BMI, FG, 2-h PPG, HbA1c, HOMA-IR, AST, ALT, triglycerides, and LDL. Results are presented in Table 3.

## 3. Discussion

The rising prevalence of NAFLD, combined with the knowledge that medical treatments would be long-term, highlighted the need for cost-effective treatments [18]. Currently, there is no approved treatment for NAFLD. Although diet modifications and regular exercise regimens are frequently recommended, they take time to accomplish results [19]. Previous results for UDCA focused on the inflammatory and fibrotic changes rather than simple steatosis [20,21,22]. The current study aims to define the key differences between empagliflozin versus UDCA in treating NAFLD in type 2 diabetic patients.

MRI-PDFF was utilized for liver fat quantification over liver histology because it has been shown that MRI-PDFF is strongly correlated with the percentage and grade of steatosis compared to ultrasonography in patients with metabolic disorders [23]. Liver biopsy is invasive, inappropriate for follow-up patients, and only recommended in patients with NASH rather than simple steatosis [24].

The present study showed that both groups achieved clinically significant reductions in hepatic steatosis. These improvements were evident on ultrasound images and MRI-PDFF maps. The EMPA group had a significantly higher LFC (%) reduction than the UDCA group. Additionally, the reductions in LFC (%) in the EMPA and UDCA groups were higher than reductions from glycemic adjustment (if any) in the placebo group (*p* < 0.0001).

The placebo group showed significant minor improvement in liver steatosis. Still, it failed to show significant LFC (%) reductions in segmental liver fat fraction mapping (except liver segments IV and VIII). Additionally, it failed to reduce liver steatosis in nearly one-third of the patients (22/80; 27.5%), and these improvements would be considered clinically insignificant. These improvements may be due to these patients’ minor weight and glycemic changes. Our cohort followed the same restricted diet and exercise regimens and optimized antidiabetic therapy to normalize the possible benefits of weight and glycemic control on LFC (%) in all patients. Therefore, the effects of our study drugs would be above the placebo group. That was consistent with a recent study that reported positive impacts on liver histology with glimepiride compared to an SGLT2 inhibitor [25].

Berberine salt of UDCA (1000mg/twice daily) decreased LFC significantly (−4.8%; *p* = 0.01); however, the low dose (500mg/twice daily) showed non-significant changes in LFC [26]. This study’s higher percentage of reduction is due to increasing the bioavailability of both molecules after ingesting the ionic salt [27]. Furthermore, empagliflozin 10 mg showed significant LFC (%) reductions as measured by Fibroscan or MRI-PDFF (*p* < 0.0001) [28,29].

BMI reductions of 5% or more contribute to reducing liver steatosis [30]. A study conducted in a primary care setting revealed that lifestyle recommendations alone were insufficient to achieve >5% BMI reduction in NAFLD patients [31]. Even though all groups followed the same diet for the study duration, the placebo group failed to show BMI changes. EMPA and UDCA had significant BMI and waist-to-hip ratio reductions. In the current study, the EMPA group had a 6.6% BMI reduction from baseline, while the UDCA group had a 7.7% reduction. UDCA (as a bile acid derivative) activates a protein-coupled bile acid receptor (TGR-5) that enhances GLP-1 secretion from intestinal L cells. Bile acid receptors, Farnesoid X receptor (FXR), and TGR5 are viable treatment options for treating metabolic disorders such as type 2 diabetes and obesity. GLP-1 enhances satiety and promotes weight loss in normal individuals and T2DM patients [32,33].

LFC (%) reductions in both groups were not correlated to BMI and waist-to-hip ratio improvements. It is worth noting that all patients included in this study had a BMI over 25 kg/m^2^. Subsequently, BMI and abdominal obesity improvements are unlikely to impact LFC.

A previous study reported that empagliflozin decreased weight, BMI, waist-to-hip ratio, liver steatosis, and fibrosis in patients with NAFLD without T2DM. In this study, they did not find any significant correlation between changes in liver steatosis and BMI [12]. Additionally, Nadinskaia et al. reported that UDCA improved fatty liver independent of weight reductions [16].

UDCA achieved better weight control than EMPA. LFC (%) reductions were observed in all patients of both groups, regardless of weight loss and BMI improvements. Six (7.5%) patients without BMI improvements in the EMPA group achieved a mean LFC (%) reduction equal to 5.9%, and eight (10%) patients in the UDCA group achieved a mean LFC (%) reduction equivalent to 3.5%.

Insulin resistance, serum insulin levels, and oxidative stress are risk factors for several Mets components, including diabetes, hypertension, cardiovascular, and NAFLD [34]. In animal models, UDCA decreased fasting glucose, insulin, and hepatic insulin resistance, concluding that UDCA is effective in treating hepatic steatosis accompanied by T2DM [35]. In a pilot randomized controlled trial, UDCA showed beneficial effects on the glycemic profile when added to sitagliptin [32]. Moreover, our study agreed with a previous study that found EMPA significantly reduced insulin resistance [36]. Another study reported a non-significant decrease in insulin resistance with EMPA [12].

A Japanese study reported a positive correlation between LFC (%) and glycemic profile changes with ipragliflozin (an SGLT2 inhibitor) [37]. In correlation analysis, the liver fat reductions in the EMPA and UDCA groups were not correlated with the improvements in fasting, 2-h PPG, HbA1c, and insulin resistance.

Simple non-invasive fibrosis scores have been validated and recommended in daily practices due to their low cost and ease of use. NFS is one of the most widely used tests for detecting advanced fibrosis (F3–F4) [38].

Compared to the placebo group, empagliflozin and UDCA therapy significantly reduced the risk of developing advanced fibrosis. The exact mechanism of action of empagliflozin in improving liver fibrosis is unclear. It is hypothesized that empagliflozin could inhibit proinflammatory cytokines such as IL-6 and TNF-α [10]. Additionally, LFC (%) reductions seen with empagliflozin would reduce chronic inflammation and enhance fibrosis regression [28]. UDCA ameliorates fibrosis through its anti-oxidant, anti-inflammatory, and anti-apoptotic properties [13].

Additionally, there was a significant difference in the FIB-4 index changes from baseline between UDCA and EMPA, respectively, which indicates that UDCA would show slightly higher benefits for liver fibrosis. NFS changes differed mathematically but not statistically. This lack of statistically significant difference may be associated with the short duration of follow-up. Furthermore, the diagnostic performance of FIB-4 is better for detecting fibrosis in various subgroups of metabolic-associated NAFLD disease [39]. Finally, the number of patients who ruled out advanced fibrosis (with both FIB-4 and NFS) to a lower degree favored UDCA over EMPA (32 vs. 26 patients). After treatment, only the UDCA group had eight patients (10%) with normal fibrosis scores.

NAFLD is connected to higher levels of triglycerides and LDL and lower HDL levels [40]. In the current study, empagliflozin and UDCA had similar trends in improving lipid parameters. Only empagliflozin achieved a significant increase in HDL. Maria et al. reported positive changes in lipid profiles with UDCA treatment [16]. UDCA reduces cholesterol production and absorption and increases bile acid synthesis [26,41]. Previous studies supported our study findings of the EMPA group, as SGLT2 inhibitors significantly decreased serum triglycerides in patients with/without diabetes [6,28,29,42].

The placebo group had insignificant increases in eGFR, reflecting the possible future deterioration of kidney function, especially when accompanied by diabetes as a risk factor. Both UDCA and EMPA groups significantly decreased eGFR. Despite that, all increases or decreases were within the predefined normal range.

Kim et al. reported that UDCA reduced ALT, AST, and GGT levels by 40.3%, 33.9%, and 23%, respectively, after four weeks [43]. Other studies found a statistically significant decrease in serum ALT, AST, and GGT with empagliflozin [11,29]. These reductions were unrelated to the patient’s glycemic status [12]. Former studies reported that SGLT2 inhibitors improved liver enzymes and GGT [5,9,11,29,44].

Liver enzymes are surrogate indicators that do not always predict or correlate with steatosis reduction [30]. During the study, the EMPA and UDCA groups had a significantly comparable reduction in liver enzymes and GGT. The mean AST and ALT reductions in the EMPA group were 38.9% versus 45.2%, respectively, and in the UDCA group, they were 45.6% versus 38.2%, respectively. The present results demonstrated no correlation between LFC (%) changes and ALT in the UDCA and EMPA groups. Furthermore, the E-LIFT study did not find any correlation between liver fat reductions with empagliflozin therapy and different study parameters [29].

The current study was conducted on patients with diabetes with poor glycemic control and different degrees of hepatic steatosis. That was evident as our study groups had higher baseline LFC (%) than reported in the literature [5,29].

Despite the encouraging results observed in our study, we could not include a liver biopsy due to its invasive nature and the low acceptance rate in patients with simple steatosis.

## 4. Materials and Methods

A randomized and double-blinded clinical study was conducted at the outpatient clinic of diabetes at Minia University Hospital. Clinicians performing laboratory tests, data analysts (biostatisticians), and radiologists were blinded to the patient’s data and allocation. This study was registered in clinicaltrials.gov (NCT04910178).

Patients above 18 years old were eligible to participate according to the following criteria: (1) confirmed diagnosis of T2DM [45], (2) using sulfonylurea (as T2DM standard of care (SOC)) for at least the previous six months, (3) having any degree of liver steatosis on ultrasound.

Ultrasound was performed to grade NAFLD patients according to the criteria mentioned in the previous literature [46].

All patients presented to the diabetes clinic of Minia University Hospital were screened for eligibility criteria. Eligible patients were asked for their voluntary informed consent to participate in the trial. Afterward, patients were divided into mild (80 patients), moderate (80 patients), and severe NAFLD (80 patients) and then randomly assigned to one of the study groups.

During the study, 305 adult patients (>18 years old) were screened, and only 240 patients completed the trial as follows:

Group I: (EMPA group): included 80 patients receiving empagliflozin (25 mg once daily) [5] added to T2DM SOC.

Group II: (UDCA group): included 80 patients receiving ursodeoxycholic acid (250 mg twice daily) [47] added to T2DM SOC.

Group III: (Placebo group): included 80 patients receiving placebo added to T2DM SOC.

The ultrasound was performed using Toshiba Xario Aplio 500 US system with a convex probe (2–5 μHz). All patients followed the same restricted diet and exercise regimen during the six-month treatment period. All patients were subjected to physical and abdominal examinations alongside an electrocardiogram and abdominal ultrasound during the screening visit. Additionally, they were asked to report diabetes onset, complications, chronic diseases, and drugs.

Physical examination of all patients and controls included blood pressure (systolic (SBP) and diastolic blood pressure (DBP) measurements), calculation of BMI and waist-to-hip ratio, and abdominal ultrasound.

Laboratory investigations included: fasting glucose (FG) and 2 hour postprandial glucose (2-h PPG) levels (mg/dL), HbA1c (%), liver enzymes (alanine aminotransferase (ALT; mg/dL), aspartate aminotransferase (AST; mg/dL), gamma-glutamyl transferase (GGT; U/L), and alkaline phosphatase (ALP; U/L)), lipid profile (triglycerides, total cholesterol, high-density lipoprotein (HDL), and low-density lipoprotein (LDL); mg/dL), serum insulin level (µIU/L).

Furthermore, changes in insulin resistance (HOMA-IR) were calculated as [48]:

$$\frac{Fasting\;insulin\;\left(\mathrm{IU}/\mathrm{mL}\right)\times Fasting\;glucose\;\left(\mathrm{mg}/\mathrm{dL}\right)}{405}$$

Additionally, beta-cell function was estimated as (HOMA-B) [48]:

$$\frac{360\times Fasting\;Insulin\;\left(\mathrm{IU}/\mathrm{mL}\right)\;}{Fasting\;glucose\left(\mathrm{mg}/\mathrm{dL}\right)-63}$$

NFS was calculated as: (−1.675 + [0.037 × age (years)] + [0.094 × BMI (kg/m^2^)] + [1.13 × impaired fasting glucose/diabetes (yes = 1, no = 0)] + [0.99 × AST/ALT ratio] − [0.013 × platelet (×10^9^ /L)] − [0.66 × albumin (g/dL)] [49]. NFS values < −1.455 indicate a low likelihood of fibrosis; NFS values between −1.455 and 0.675 indicate an indeterminate fibrosis probability, and NFS values > 0.675 refer to the high likelihood of fibrosis. 

Moreover, the FIB-4 index was calculated as (Age [yr.] × AST [U/L])/(PLT [10^9^/L] × ALT [U/L]^1/2^). Patients with fibrosis stages 0–1, 2–3, and 4–6 have FIB-4 index values of 1.45, 1.45–3.25, and >3.25, respectively [50].

All outcomes were collected at baseline and after six months. Moreover, safety was assessed through vital signs, the incidence of adverse events, physical examination, and abnormalities in blood chemistry. Additionally, the estimated glomerular filtration rate (eGFR; mL/min/1.73m^2^) was calculated according to the CKD-EPI 2021 equation [51].

### 4.1. MRI-PDFF Protocol

The primary outcome was estimating LFC (%) changes using the mDixon Quant. technique [46,52]. Liver fats were quantified using a 1.5-T MRI system (Philips MR system Ingenia). This technique yields in-phase (IP) and out-of-phase (OP) scans (separating water and fat signals). These images were used to calculate LFC (%) according to this equation: [(SI_IP_ − SI_OP_)/2SI_IP_] ×100. 

SIIP and SIOP refer to the hepatic-to-splenic SI ratios in the IP and OP images. SI was estimated as the mean regions of interest (ROIs) placed in each liver segment (Segment I, II, III, IVa, IVb, V, VI, VII, and VIII) and spleen, avoiding major vessels and bile ducts, visual artifacts, and organ margins. The previous steps were performed by a radiologist, who was blinded to the clinical data of study patients [29,53].

Patients were classified according to the following MRI-PDFF values: no steatosis (<6.5%), Grade I (>6.5 and <17.4%), Grade II (>17.4 and <22.1%), and Grade III (>22.1%) [52].

### 4.2. Exclusion Criteria

Those with type 1 diabetes or ketoacidosis, heavy alcohol consumers, end-stage organ failure, chronic renal failure (estimated eGFR below 60 mL/min/1.73 m^2^, CrCl below 60 mL/min, or on dialysis), liver diseases (e.g., viral hepatitis, drug-induced liver disease, hepatocellular carcinoma, hepatobiliary disease, or autoimmune hepatitis), cardiac disease (especially NYHA classes III/IV), eating disorders or having previous bariatric surgery, immunocompromised patients or with a history of inflammatory (acute or sclerosing cholangitis), immunological, or malignant diseases, and pregnant or lactating females were excluded from the study. Moreover, patients having any contraindication or hypersensitivity to study drugs or MRI procedures (cardiac pacemakers or implanted devices with ferromagnetic fields) were excluded.

### 4.3. Sample Size Calculation

Based on the previous clinical studies [29,54], anticipating a 5% reduction or more in LFC (%) would be clinically acceptable. Upon these assumptions and accounting for dropouts, 80 patients per group were required to achieve a power of 80%, at least at a significance level of 0.05. 

### 4.4. Statistical Analysis

Data were tabulated and analyzed using the statistical package SPSS software (Version 25.0, SPSS Inc., Chicago, IL, USA). Categorical data were analyzed using the X2 test to compare the baseline and post-treatment data and expressed as n (%). Paired student t-test was used to compare two means for the same group. Continuous variables were reported as mean ± SD and 95% confidence interval of the difference (95% C.I). Bivariate correlation between the primary outcome and other study outcomes was done using Pearson correlation. One-way analysis of variance (ANOVA) (for all groups) followed by a post hoc test was used to compare the statistical significance of baseline values. Additionally, repeated measure ANOVA was done to indicate multiple time differences in liver enzymes. *p*-values < 0.05 were reported as statistically significant.

## 5. Conclusions

Treating NAFLD in diabetic patients with EMPA would exhibit a greater liver steatosis regression, better glycemic profile, and serum triglycerides reduction than UDCA. However, UDCA improved liver fibrosis scores and insulin resistance more than EMPA. Both drugs were comparable in decreasing liver enzymes and BMI. We could sum up these findings to suggest that both EMPA and UDCA could be used safely and effectively for NAFLD patients with diabetes. Further investigations should be done to confirm these findings for the whole population, especially diabetic patients with concomitant diseases.

## Figures and Tables

**Table 1:** Changes in biochemical characteristics after six months.

Parameters		EMPA	UDCA	Placebo
SBP (mmHg)	Baseline (mean ± SD)	122.50 ± 8.50	120.75 ± 7.30	129.75 ± 14.18
	Post-treatment (mean ± SD)	117.00 ± 5.71	116.25 ± 3.93	123.75 ± 4.66
	Difference (95% C.I)	−5.50 (−9.72, −1.28)	−4.50 (−7.80, −1.20)	−6.00 (−10.90, −2.10)
	*p*-value	0.01	0.01	0.001
DBP (mmHg)	Baseline (mean ± SD)	81.50 ± 6.50	77.25 ± 6.78	83.00 ± 8.94
	Post-treatment (mean ± SD)	75.75 ± 5.20	73.75 ± 4.25	80.25 ± 3.43
	Difference (95% C.I)	−5.75 (−8.61, −2.88)	−3.50 (−6.35, −0.65)	−2.75 (−6.01, 0.51)
	*p*-value	0.0001	0.01	0.094
BMI (Kg/m^2^)	Baseline (mean ± SD)	32.57 ± 4.30	33.52 ± 4.87	33.90 ± 5.82
	Post-treatment (mean ± SD)	30.42 ± 3.64	30.95 ± 4.11	34.10 ± 5.41
	Difference (95% C.I)	−2.15 (−2.79, −1.51)	−2.57 (−3.47, −1.67)	0.20 (−0.65, 1.04)
	*p*-value	0.0001	0.0001	0.633
Waist-to-hip ratio	Baseline (mean ± SD)	0.946 ± 0.06	0.972 ± 0.10	0.969 ± 0.04
	Post-treatment (mean ± SD)	0.932 ± 0.06	0.942 ± 0.09	0.966 ± 0.05
	Difference (95% C.I)	−0.01 (−0.02, −0.01)	−0.03 (−0.05, −0.01)	−0.003 (−0.02, 0.01)
	*p*-value	0.0001	0.001	0.527
AST (U/L)	Baseline (mean ± SD)	29.50 ± 16.86	33.39 ± 20.47	25.85 ± 9.65
	Post-treatment (mean ± SD)	18.00 ± 4.01	18.15 ± 6.38	29.25 ± 11.71
	Difference (95% C.I)	−11.50 (−18.97, −4.02)	−15.24 (−23.05, −7.42)	3.40 (−1.80, 4.81)
	*p*-value	0.004	0.001	0.001
ALT (U/L)	Baseline (mean ± SD)	28.75 ± 14.26	31.60 ± 21.37	26.05 ± 10.60
	Post-treatment (mean ± SD)	15.75 ± 4.02	19.50 ± 10.16	30.40 ± 11.18
	Difference (95% C.I)	−13.00 (−18.87, −7.13)	−12.10 (−20.93, −3.27)	4.35 (0.78, 9.91)
	*p*-value	0.0001	0.0001	0.187
ALP (U/L)	Baseline (mean ± SD)	112.60 ± 46.85	121.75 ± 41.97	80.75 ± 24.03
	Post-treatment (mean ± SD)	113.19 ± 90.29	101.05 ± 89.32	80.95 ± 31.41
	Difference (95% C.I)	0.59 (−53.21, 54.38)	−20.70 (−63.07, 21.66)	0.20 (−11.76, 12.16)
	*p*-value	0.982	0.319	0.024
GGT (U/L)	Baseline (mean ± SD)	47.83 ± 16.06	46.03 ± 14.09	48.02 ± 12.65
	Post-treatment (mean ± SD)	32.65 ± 12.96	28.20 ± 7.95	44.85 ± 11.54
	Difference (95% C.I)	−15.18 (−22.68, −7.66)	−17.83 (−24.59, −11.07)	−3.17 (−9.79, 3.45)
	*p*-value	0.0001	0.0001	0.972
FG (mg/dL)	Baseline (mean ± SD)	169.95 ± 39.26	152.20 ± 57.75	138.00 ± 43.16
	Post-treatment (mean ± SD)	121.90 ± 29.20	112.45 ± 29.92	112.15 ± 16.58
	Difference (95% C.I)	−48.05 (−65.51, −30.59)	−39.75 (−57.83, −21.67)	−25.85 (−43.14, −8.56)
	*p*-value	0.0001	0.0001	0.0001
2-h PPG2-h PPG(mg/dL)	Baseline (mean ± SD)	316.00 ± 96.02	258.15 ± 57.74	225.25 ± 69.34
	Post-treatment (mean ± SD)	190.15 ± 37.62	169.70 ± 40.30	177.45 ± 40.76
	Difference (95% C.I)	−125.85 (−169.92, −81.78)	−88.45 (−114.80, −62.11)	−47.80 (−73.59, −22.01)
	*p*-value	0.0001	0.0001	0.001
HbA1c (%)	Baseline (mean ± SD)	8.97 ± 1.39	8.54 ± 1.50	7.98 ± 1.18
	Post-treatment (mean ± SD)	7.25 ± 0.42	7.40 ± 0.56	7.37 ± 0.43
	Difference (95% C.I)	−1.72 (−2.23, −1.21)	−1.14 (−1.65, −0.62)	−0.61 (−1.06, −0.17)
	*p*-value	0.0001	0.0001	0.0001
HOMA-IR	Baseline (mean ± SD)	7.25 ± 6.41	6.57 ± 6.22	6.23 ± 4.02
	Post-treatment (mean ± SD)	4.30 ± 3.19	3.31 ± 2.97	5.43 ± 2.52
	Difference (95% C.I)	−2.95 (−5.40, −0.47)	−3.26 (−5.75, −0.76)	−0.80 (−2.42, 0.65)
	*p*-value	0.02	0.01	0.262
HOMA-B	Baseline (mean ± SD)	65.26 ± 57.17	88.05 ± 56.41	98.99 ± 52.97
	Post-treatment (mean ± SD)	119.46 ± 103.55	107.59 ± 92.53	112.46 ± 68.92
	Difference (95% C.I)	54.20 (4.88, 103.53)	19.54 (−15.20, 54.29)	13.47 (−26.38, 40.55)
	*p*-value	0.03	0.319	0.210
Insulin (μIU/L)	Baseline (mean ± SD)	17.36 ± 14.95	16.94 ± 11.73	17.78 ± 9.71
	Post-treatment (mean ± SD)	14.30 ± 9.76	11.61 ± 9.19	19.40 ± 8.62
	Difference (95% C.I)	−3.06 (−8.31, 2.20)	−5.33 (−10.70, 0.04)	1.62 (−1.58, 4.81)
	*p*-value	0.238	0.051	0.304
eGFR (mL/min/1.73m^2)^	Baseline (mean ± SD)	86.01 ± 23.27	81.39 ± 19.42	97.25 ± 33.33
	Post-treatment (mean ± SD)	88.50 ± 27.37	90.98 ± 24.71	85.32 ± 28.23
	Difference (95% C.I)	2.49 (−11.33, 16.30)	9.95 (−1.74, 20.93)	−11.93 (−23.58, −0.28)
	*p*-value	0.710	0.092	0.045

Paired *t*-test. Data are presented as mean ± standard deviation, significant if *p*-value < 0.05. DBP = diastolic blood pressure, SBP = systolic blood pressure, BMI = body mass index, FG = fasting glucose, 2-h PPG = 2 hour postprandial glucose, HbA1c = glycosylated hemoglobin, HOMA-B = hemostatic model assessment for β-cell function, HOMA-IR = hemostatic model assessment for insulin resistance, AST = serum aspartate transaminase, ALT = serum alanine transaminase, ALP = alkaline phosphatase, GGT = gamma-glutamyl transferase, C.I = confidence interval, eGFR = estimated glomerular filtration rate.

**Table 2:** Full liver fat fraction mapping by MRI-PDFF in study groups.

Liver Segments	EMPA			UDCA			Placebo		
	Baseline	Post-Treatment	*p*-Value	Baseline	Post-Treatment	*p*-Value	Baseline	Post-Treatment	*p*-Value
I	18.57 ± 9.52	10.64 ± 5.56	0.001	19.19 ± 8.58	20.72 ± 8.09	0.585	19.91 ± 8.87	19.18 ± 9.39	0.572
II	23.83 ± 9.09	12.38 ± 5.28	0.0001	20.51 ± 6.97	17.57 ± 6.20	0.108	19.47 ± 8.72	17.34 ± 7.54	0.074
III	20.59 ± 6.90	13.24 ± 5.30	0.0001	18.67 ± 8.17	20.92 ± 7.26	0.216	20.29 ± 8.19	17.72 ± 8.91	0.026
IVa	23.16 ± 7.15	14.14 ± 6.08	0.0001	21.84 ± 8.07	21.51 ± 8.95	0.779	18.61 ± 8.83	17.58 ± 8.97	0.294
IVb	20.84 ± 8.11	13.68 ± 7.97	0.001	19.89 ± 8.78	13.01 ± 7.90	0.048	19.49 ± 7.81	16.86 ± 7.78	0.051
V	24.16 ± 9.82	14.25 ± 7.20	0.001	15.90 ± 5.95	10.58 ± 6.05	0.042	19.52 ± 8.44	17.58 ± 9.04	0.099
VI	23.18 ± 8.72	13.02 ± 6.53	0.001	13.39 ± 5.09	14.71 ± 7.40	0.604	19.47 ± 7.07	17.26 ± 7.11	0.04
VII	23.20 ± 7.89	12.20 ± 5.99	0.001	13.25 ± 4.69	13.56 ± 7.90	0.903	20.73 ± 6.49	19.11 ± 6.49	0.112
VIII	22.37 ± 10.68	12.76 ± 6.80	0.002	19.34 ± 8.42	13.61 ± 7.07	0.017	19.59 ± 7.93	17.59 ± 7.57	0.003
Total LFC (%)	21.54 ± 7.29	12.80 ± 5.40	0.0001	19.96 ± 6.58	14.24 ± 7.10	0.0001	19.91 ± 7.25	17.92 ± 7.62	0.006

Paired *t*-test. Significant if *p*-value < 0.05. LFC = liver fat content, MRI-PDFF = magnetic resonance imaging-proton density fat fraction. Data are presented as: mean ± standard deviation.

**Table 3:** Correlation study between changes in LFC (%) and changes in selected measured parameters after six months of treatment in study groups.

	∆ LFC (%)	EMPA		UDCA		Placebo	
Parameters		r	*p*-Value	r	*p*-Value	r	*p*-Value
∆ BMI		0.082	0.730	0.067	0.779	0.474	0.035
∆ FG		0.163	0.491	−0.004	0.988	0.341	0.141
∆ 2-h PPG		0.160	0.500	−0.256	0.276	0.542	0.014
∆ HbA1c		−0.59	0.806	0.113	0.636	0.128	0.590
∆ HOMA-IR		0.274	0.242	0.081	0.731	0.252	0.283
∆ AST		−0.346	0.135	−0.383	0.095	−0.546	0.013
∆ ALT		−0.258	0.272	−0.195	0.410	−0.296	0.206
∆ Triglycerides		0.403	0.057	0.037	0.876	0.193	0.416
∆ LDL		0.314	0.178	0.009	0.969	−0.481	0.032

Pearson correlation. Significant if *p*-value < 0.05. r = correlation coefficient, LFC = liver fat content, BMI = body mass index, FG = fasting glucose, 2-h PPG = 2 h postprandial glucose, HbA1c = glycosylated hemoglobin, HOMA-IR = hemostatic model assessment for insulin resistance, AST = serum aspartate transaminase, ALT = serum alanine transaminase, LDL = low density lipoprotein.

## Data Availability

Data is contained within the article and Supplementary Material.

## Supplementary Materials

The following supporting information can be downloaded at: https://www.mdpi.com/article/10.3390/ph15121516/s1, Figure S1: Changes in liver enzymes after 3 and 6-months posttreatment; Table S1: Demographic data of study groups; Table S2: Baseline characteristics of study patients.
