# Supplementary material for: The Impact of an SGLT2 Inhibitor versus Ursodeoxycholic Acid on Liver Steatosis in Diabetic Patients
Source: Pharmaceuticals (Basel). 2022 Dec 5;15(12):1516. doi: 10.3390/ph15121516 (PMC9786599; doi:10.3390/ph15121516)
Supplement: Supplementary file 1 [file pharmaceuticals-15-01516-s001.zip › pharmaceuticals-2052680-supplementary.pdf]

Supplementary Table S1. Demographic data of study groups.

Variables		EMPA group	UDCA group	Control group	*p*-value
Gender	Male	27 (33.75%)	25 (31.25%)	25 (31.25%)	0.671
	Female	53 (66.25%)	55 (68.75%)	55 (68.75%)	
Age	Range	30-66	30-65	29-62	0.432
	Mean±SD	47.75±8.59	49.49±6.66	47.36±8.55	
BMI	Range	23-42	25-42	26-42	0.397
	Mean±SD	32.67±4.34	33.48±4.75	32.02±5.68	
DM comorbidities	Hypertension	25 (31.3%)	31 (38.8%)	35 (43.8%)	0.276
	Dyslipidemia	19 (23.8%)	20 (25.0%)	22 (27.5%)	0.770
	Neuropathy	47 (58.8%)	32 (40.0%)	41 (51.3%)	0.150
Medications	Statins	19 (23.8%)	20 (25.0%)	22 (27.5%)	0.583
	B-blockers	13 (16.3%)	14 (17.5%)	12 (15.0%)	0.803
	ACEI's/ARBs	6 (7.5%)	11 (13.8%)	20 (25.0%)	0.017
	Diuretics	10 (12.5%)	10 (12.5%)	6 (7.5%)	0.338
	NSAID's	20 (25.0%)	19 (23.8%)	24 (30.0%)	0.410
	Pregabalin/ Gabapentin	20 (25.0%)	15 (18.8%)	12 (15.0%)	0.328
	Vitamin B12 injections	45 (56.3%)	37 (46.3%)	50 (62.5%)	0.183

BMI = body mass index, SD = standard deviation, ACEI's = angiotensin competitive enzyme inhibitors, ARB = angiotensin receptor blocker, NSAID = nonsteroidal anti-inflammatory drug, DM = diabetes mellitus.

Supplementary Table S2. Baseline characteristics of study patients.

Parameters		EMPA	UDCA	CONTROL	P1	P2	P3	P4
SBP (mmHg)	Mean±SD	122.50±8.50	120.75±7.30	129.75±14.18	0.02**	0.080	0.023	0.857
DBP (mmHg)	Mean±SD	81.50±6.50	77.25±6.78	83.00±8.94	0.049**	0.803	0.048	0.181
BMI (Kg/m^2)	Mean±SD	32.57±4.30	33.52±4.87	33.90±5.82	0.695	0.686	0.970	0.822
Waist-to-hip ratio	Mean±SD	0.946±0.06	0.972±0.10	0.969±0.04	0.469	0.574	0.992	0.502
FG (mg/dL)	Mean±SD	169.95±39.26	152.20±57.75	138.00±43.16	0.111	0.092	0.613	0.467
2hr-PPG (mg/dL)	Mean±SD	316.00±96.02	258.15±57.74	225.25±69.34	0.002**	0.001	0.365	0.050
HbA1c (%)	Mean±SD	8.97±1.39	8.54±1.50	7.98±1.18	0.080	0.065	0.403	0.583
HOMA-IR	Mean±SD	7.25±6.41	6.57±6.22	6.23±4.02	0.847	0.838	0.980	0.925
HOMA-B	Mean±SD	65.26±57.17	88.05±56.41	98.99±52.97	0.156	0.142	0.808	0.403
Insulin (µIU/L)	Mean±SD	17.36±14.95	16.94±11.73	17.78±9.71	0.977	0.994	0.974	0.993
LFC (%)	Mean±SD	21.54±7.29	19.96±6.59	19.91±7.26	0.710	0.748	0.999	0.760
NFS	Mean±SD	-0.96±0.98	-0.84±1.14	-1.53±1.26	0.131	0.260	0.142	0.940
FIB-4	Mean±SD	1.03±0.66	1.20±0.67	0.80±0.29	0.02**	0.091	0.001	0.260
AST (U/L)	Mean±SD	29.50±16.86	33.39±20.47	25.85±9.65	0.350	0.760	0.316	0.723
ALT (U/L)	Mean±SD	28.75±14.26	31.60±21.37	26.05±10.60	0.553	0.856	0.522	0.841
ALP (U/L)	Mean±SD	112.60±46.85	121.75±41.97	80.75±24.03	0.004**	0.032	0.004	0.738
GGT (U/L)	Mean±SD	47.83±16.06	46.03±14.09	48.02±12.65	0.890	0.999	0.899	0.917
Total cholesterol (mg/dL)	Mean±SD	237.85±37.99	227.80±61.08	152.10±28.56	0.0001**	0.0001	0.0001	0.758
Triglycerides (mg/dL)	Mean±SD	191.00±92.09	187.75±77.33	140.35±32.02	0.052	0.075	0.989	0.102
LDL (mg/dL)	Mean±SD	152.45±28.10	154.70±34.77	93.55±24.02	0.0001**	0.0001	0.0001	0.968
HDL (mg/dL)	Mean±SD	44.80±7.25	40.65±9.42	35.75±8.13	0.005**	0.003	0.159	0.264

One-Way ANOVA. **Significant if *p*-value < 0.05. P1 = between groups, P2 = between control and empagliflozin, P3 = between control and ursodeoxycholic acid, P4 = between empagliflozin and ursodeoxycholic acid. DBP = hemoglobin, HOMA-B = hemostatic model assessment for β-cell function, HOMA-IR = hemostatic model assessment for insulin resistance, LFC = liver fat content, NFS = NAFLD fibrosis score, AST = serum aspartate transaminase, ALT = serum alanine transaminase, ALP = alkaline phosphatase, GGT = gamma glutamyl transferase, LDL =low density lipoprotein, HDL = high density lipoprotein, eGFR = estimated glomerular filtration rate, SD = standard deviation.

A) Changes in AST

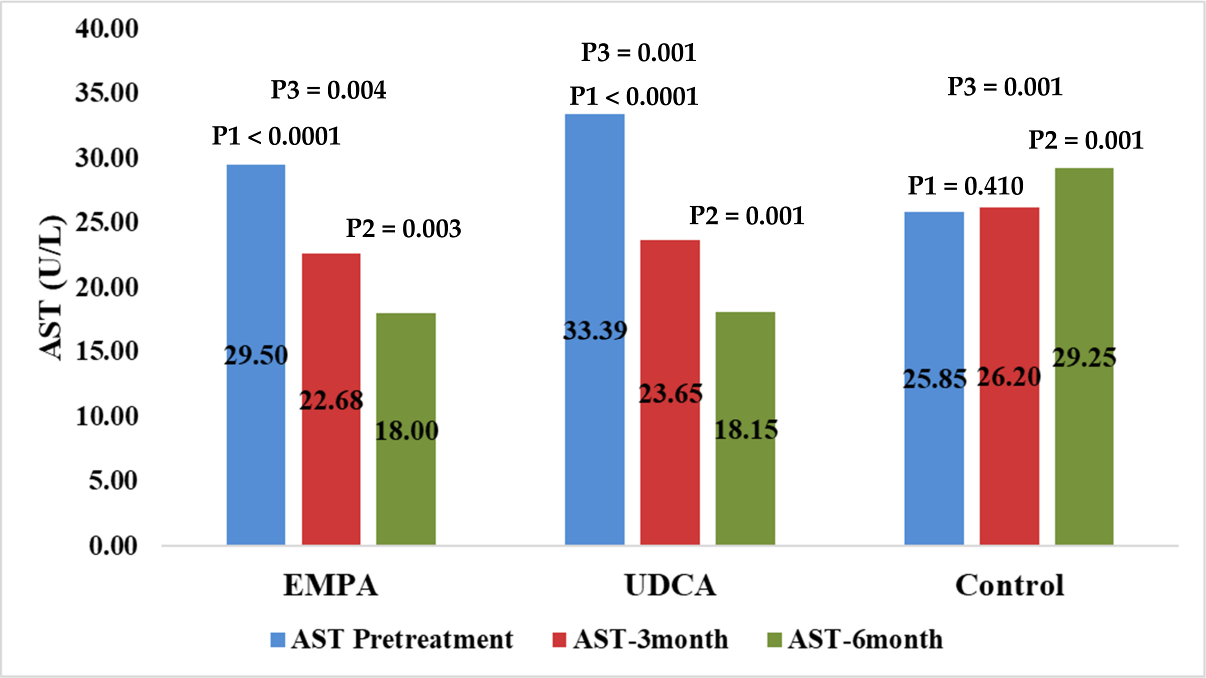

AST = Aspartate transaminase, P1 = *p*-value between pretreatment and 3-month posttreatment, P2 = *p*-value between 3-month posttreatment and 6-month posttreatment, P3 = *p*-value between pretreatment and 6-month posttreatment

B) Changes in ALT

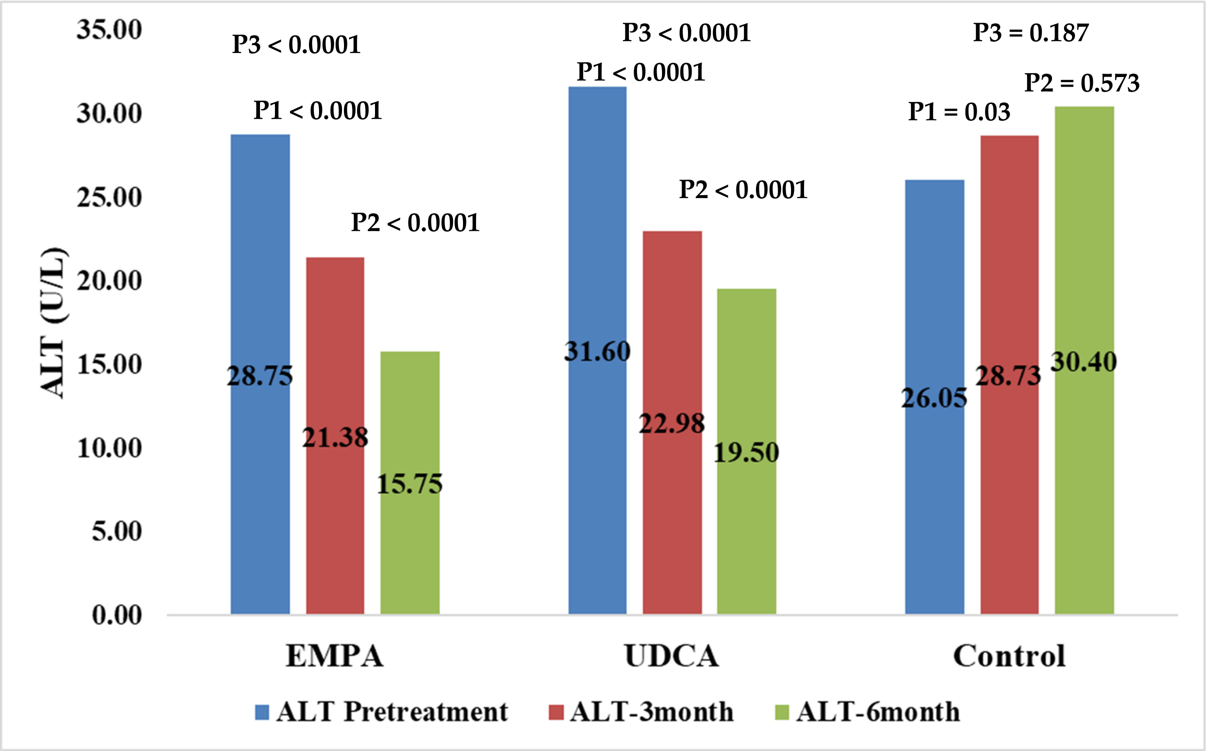

ALT = alanine transaminase, P1 = p-value between pretreatment and 3-month posttreatment, P2 = p-value between 3-month posttreatment and 6-month posttreatment, P3 = p-value between pretreatment and 6-month posttreatment

Supplementary Figure S1. Changes in liver enzymes after 3 and 6-months posttreatment.
